# Oncogene FOXK1 enhances invasion of colorectal carcinoma by inducing epithelial-mesenchymal transition

**DOI:** 10.18632/oncotarget.9457

**Published:** 2016-05-19

**Authors:** Yao Wu, Ying Peng, Meiyan Wu, Wenjing Zhang, Mengnan Zhang, Ruyi Xie, Pei Zhang, Yang Bai, Jinjun Zhao, Aimin Li, Qingzhen Nan, Ye Chen, Yuexin Ren, Side Liu, Jide Wang

**Affiliations:** ^1^ Guangdong Provincial Key Laboratory of Gastroenterology, Department of Gastroenterology, Nanfang Hospital, Southern Medical University, Guangzhou, 510515, China; ^2^ Department of Rheumatism, Nanfang Hospital, Southern Medical University, Guangzhou, 510515, China; ^3^ Department of Medical Oncology, the First People's Hospital of Yunnan Province, Kunming University of Science and Technology, Kunming, 650032, China; ^4^ Department of Gastroenterology, The First Affiliated Hospital of Nanchang University, Nanchang, 330006, China

**Keywords:** FOXK1, colorectal cancer, metastasis, epithelial-mesenchymal transition, invasion

## Abstract

Transcriptional factor FOXK1 is a member of the FOX family, involved in the cell growth and metabolism. The higher expression of FOXK1 leads to a variety of diseases and may play an important role in the development of various tumors. However, the role of FOXK1 in the progression of colorectal cancer (CRC) remains unknown. We demonstrated that FOXK1 was overexpressed in 16 types of solid tumor tissues via tissue multi-array (TMA). We found that FOXK1 induced elevated expressions and transactivities of five major oncogenes in CRC. Moreover, the elevated expression of FOXK1 was showed to be correlated with tumor progression and was a significant predictor of overall survival in CRC patients. Furthermore, it was showed that the depletion of FOXK1 expression could inhibit the migratory and invasive abilities of CRC cells. In contrast, ectopic expression of FOXK1 elicited the opposite effects on these phenotypes *in vitro*. FOXK1 promoted tumor metastasis through EMT program induction. In addition, TGF-β1 induced FOXK1 expression in a time-dependent pattern and the knockdown of FOXK1 inhibited TGF-β1-induced EMT. *In vivo*, higher expression of FOXK1 promotes CRC cell invasion and metastasis, and induces EMT in CRC as well. Alltogether, it was concluded that the higher expression of FOXK1 could indicate a poor prognosis in CRC patients since that FOXK1 induces EMT and promotes CRC cell invasion *in vitro* and *in vivo*.

## INTRODUCTION

The forkhead box (Fox) gene family is a group of highly conserved transcription factors that are expressed in diverse species, including yeast and humans. [1–3] Many FOX protein members have been documented to play critical roles in embryonic development [4] as well as organogenesis [5] and are also involved in the regulation of a variety of physiological processes [6, 7], such as metabolic processes [8], cell signaling [9], and the cell cycle [10]. Consequently, dysregulation of the functions, subcellular localization and expression of FOX transcription factors leads to the development and progression of diseases, in particular, cancer [11–13].

Forkhead box k1 (FOXK1) is a transcription factor that belongs to the forkhead family consisting of the winged-helix DNA-binding domain and the N-terminal and C-terminal transcriptional domains [14]. Yang et al. have showed that MNF/Foxk1, which mediates its DNA binding, recognizes the DNA sequence motif, WRTAAAAYA and regulates the p21, c-myc, or cdc2 gene [15]. Previous studies have suggested that knockdown of FOXK1 eliminated cell cycle-dependent oscillations and resulted in decreased cell proliferation rates and the development of the malignant phenotype in human osteosarcoma U2OS cells [16]. The data indicate a role for human FOXK1 in regulating the developmental process as well as the potential involvement of FOXK1 in tumorigenesis [17, 18].

Epithelial-mesenchymal transition (EMT), which plays an essential role in tumor invasion and metastasis [19, 20], is an essential phenotypic event during embryonic development, tissue remodeling and wound healing. EMT is also a reversible process that often occurs at the invasive front of many metastatic cancers [21]. The epithelial markers that decrease during EMT include E-cadherin and β-catenin, whereas the increased mesenchymal markers include vimentin and snail. EMT is physiologically initiated by certain autocrine factors, with TGF-β being the strongest inducer that functions in the majority of epithelial cell types tested *in vivo* [22]. Others have found that the Fox gene family and EMT play important roles in cancer metastasis [23–25]. However, the role of FOXK1 proteins in colon cancer development and progression remains unknown.

In the present study, we found that FOXK1 is highly expressed in 16 types of solid tumor tissues and that increased FOXK1 expression significantly correlated with progression, metastasis, and poor outcome in patients with colorectal cancer (CRC). Furthermore, these findings uncovered the role of FOXK1 in CRC invasion, metastasis and EMT in nude mice. The results from this study demonstrated for the first time that FOXK1 expression promoted the development of invasive properties of CRC cells.

## RESULTS

### Cancer cells expressed higher levels of FOXK1

Using a FOXK1- specific antibody in tissue specimens, we analyzed FOXK1 expression patterns using TMAs in sixteen normal or solid tumor tissues of human. Normal tissues of skin, testis and kidney showed weakly positive expression, 13 tissues were negative, including the stomach, small intestine, large intestine, rectum, and gastrointestinal tissues (Supplementary Figure 1A). However, all cancerous tissues showed positive staining (Supplementary Figure 1B).

**Figure 1:**
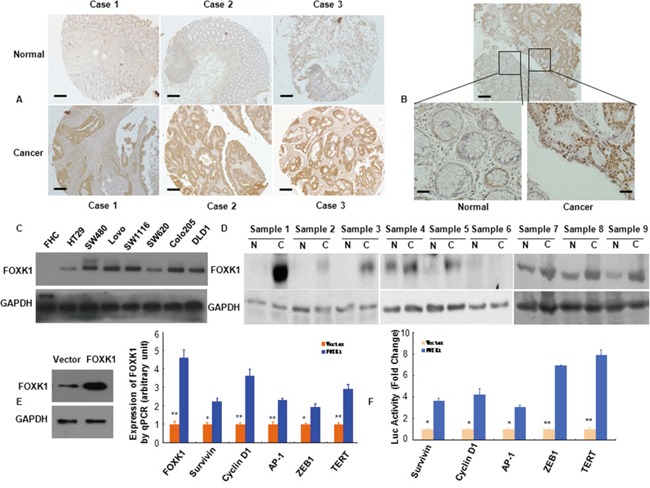
FOXK1 expression in CRC were higher than normal cells and increased multiple oncogenes expression **A, B.** FOXK1 expression in normal and malignant human colorectal tissues was detected by TMAs and IHC. **C.** Whole lysates of FHC, HT29, SW480, LoVo, SW1116, SW620, Colo205 and DLD1 were collected, and FOXK1 was detected by Western blot. GAPDH was used as the internal control (GAPDH: glyceraldehyde-3- phosphate dehydrogenase). **D.** Proteins isolated from resected tumors and adjacent non-tumorous tissue specimens were subjected to Western blotting analysis. T, CRC tissues: N, normal tissues. **E.** Expression of multiple oncogenes in stable transfectants of SW480/Vector, SW480/FOXK1 as detected by Western blot and qRT-PCR in SW480 cells. *, P < 0.05; **, P < 0.01. **F.** Luciferase (Luc) reporter constructs contain the Survivin, cyclin D1, AP-1, and ZEB1, TERT promoter of a luciferase gene in FOXK1 transfection experiments. *, P < 0.05. Scale bars, 100 μm in A; 50 μm in B.

Next, we confirmed FOXK1 expression by immunohistochemistry in excised tissues of colon or rectal in 93 CRC patients, who were from Surgery of Nanfang Hospital, Southern Medical University. We found that FOXK1-positive signals were strongly expressed in the carcinoma cells and only expressed in the carcinoma cells of all CRC samples as exemplified in Figure 1B. On the contrary, normal colon tissues did not express FOXK1 protein (Figure 1B)

Based on Western blotting, we demonstrated increased FOXK1 expression in the following seven CRC cell lines: HT-29, SW480, LoVo, SW1116, SW620, Colo205 and DLD1, compared with the normal colon cell line (FHC) (Figure 1C). We then measured FOXK1 expression in 9 pairs of matched colon normal (N) and cancerous (T) tissues by Western blot. Of the 9 cancerous tissues, 8 expressed higher levels of FOXK1 than the normal tissues (Figure 1D).

These findings demonstrated that FOXK1 was overexpressed in CRC cells and tissues.

### FOXK1 increased the expression of oncogenes

To establish stable transfectants, FOXK1 plasmids were successfully transfected into the SW480 cell line. FOXK1 overexpression was confirmed by western blot and qRT-PCR analysis (Figure 1E). We screened for potential target genes by examining the expression of 5 major oncogenes that are known to be involved in proliferation and transformation after ectopic FOXK1 expression in SW480. The mRNA expression of Survivin, Cyclin D1, AP-1, ZEB1, TERT and FOXK1 was up-regulated in stable FOXK1 transfectants (Figure 1E).

Next, we cloned the promoter region (>3000 bp) of human Survivin, Cyclin D1, AP-1 ZEB1 and TERT upstream [44, 45] of a luciferase gene in a reporter plasmid and then co-transfected with the FOXK1 cDNA construct. FOXK1 overexpression increased the luciferase activity from the reporter plasmid, which was driven by the Survivin, Cyclin D1, AP-1, ZEB1 and TERT promoter regions, by 3.66-fold, 4.22-fold, 3.0-fold, 6.90-fold and 7.87-fold, respectively (Figure 1F). Thus, FOXK1 increased the expressions and transactivities of oncogenes in CRC.

### Increased FOXK1 expression correlated with tumor progression and poor prognosis of CRC patients

**Figure 2:**
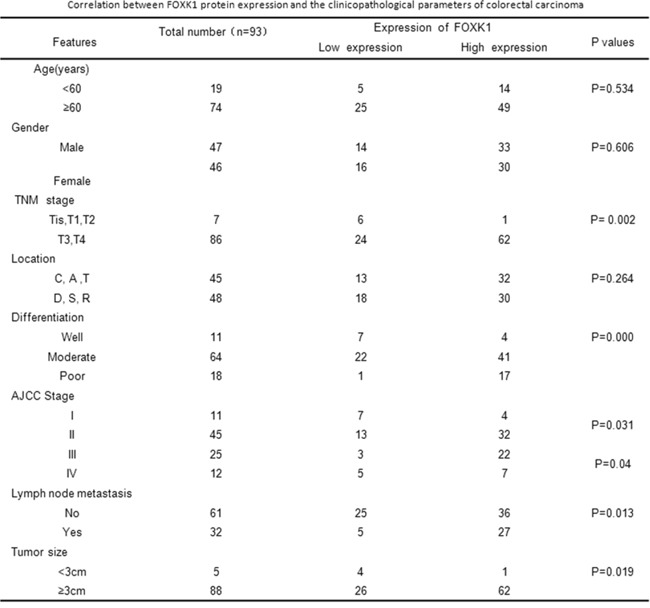
Correlation between FOXK1 protein expression and the clinicopathological parameters of colorectal carcinoma

To evaluate the relationship between FOXK1 protein and CRC progression, we analyzed the correlation between high FOXK1 expression and clinicopathological features of CRC; the data are summarized in Figure 2. No significant association was observed between FOXK1 expression and age (P = 0.534), gender (P = 0.606) and location (P = 0.264). However, FOXK1 expression significantly correlated with TNM stage (P = 0.002), differentiation (P = 0.000), AJCC Stage I/II (P = 0.031), AJCC Stage III/IV (P = 0.04), tumor size (P = 0.019) and lymph node metastasis (P = 0.013).

**Figure 3:**
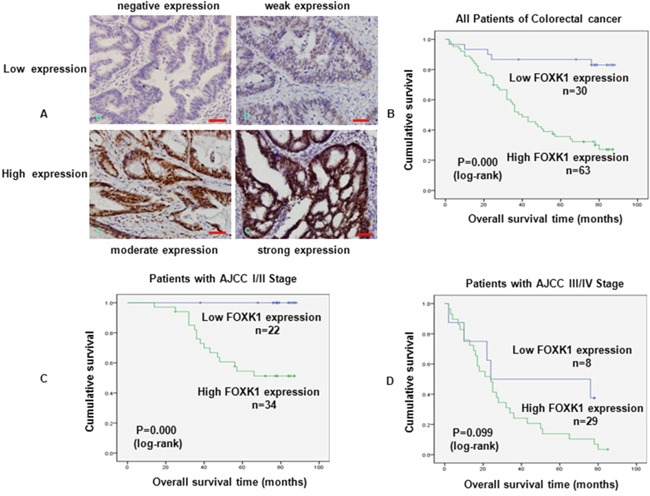
FOXK1 expression in CRC was associated with poor prognosis **A.** Expression analysis of FOXK1 protein in CRC by immunohistochemistry. Immunoreactivity in FOXK1 staining was localised in the nucleus. (a) negative expression of FOXK1 in CRC; (b) weak expression of FOXK1 in CRC; (c) moderate expression of FOXK1 in CRC; (d) strong expression of FOXK1 in CRC. **B.** Kaplan -Meier survival analysis of overall survival in all patients, **C.** patients at the early stage of CRC and **D.** patients at the late stage of CRC according to FOXK1 expression. The log-rank test was used to calculate P values. Scale bars, 100 μm in A.

Next, we evaluated the prognostic effect of FOXK1 on overall survival by comparing the overall survival of CRC patients with high or low FOXK1 protein levels (Figure 3A). Of the 93 surgical CRC specimens, 63 cases exhibited a high expression of FOXK1, whereas low expression was found in the other 30 cases. Among the participants, patients with high FOXK1 expression were associated with a significantly lower 7-year survival rate than those with a low expression, according to Kaplan-Meier curve assessment (P = 0.000, log-rank test; Figure 3B). Such a relationship observed in patients with early-stage CRC (*i.e.*, American Joint Committee on Cancer (AJCC) stage I and II; P = 0.000, Figure 3C) was more obvious than that in late-stage CRC (*i.e.*, AJCC stage III and IV; P = 0.099, Figure 3D). Unfortunately, the prognostic value of FOXK1 expression in selective patient subgroups stratified according to AJCC stage was not evident.

The above findings suggested that an increased FOXK1 expression was significantly correlated with progression, metastasis, and poor outcome in CRC patients.

### The knockdown of FOXK1 suppressed CRC cell metastasis and invasion

**Figure 4:**
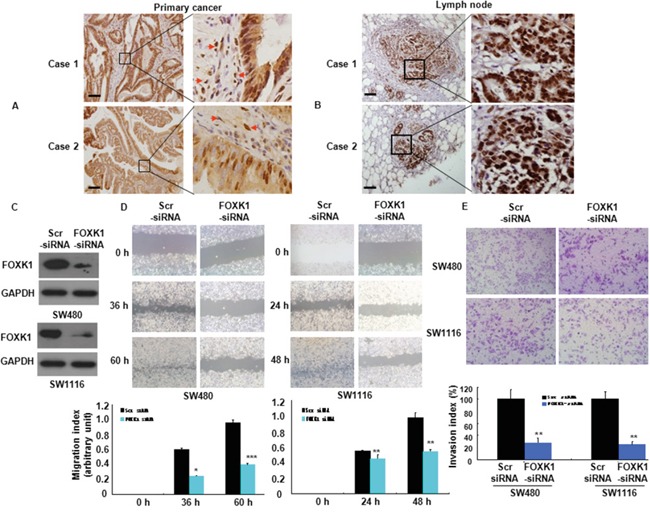
FOXK1 was associated with the invasive and metastatic potential of CRC **A, B.** The representative results of IHC staining for FOXK1 of CRC tissues with the primary and lymph node metastatic sites. Arrow, stroma cells. **C.** FOXK1 expression was detected by Western blot. **D.** Images of the wound closure of monolayer SW480 and SW1116 transfected with siRNA. *, P < 0.05; **, P < 0.01, ***, P < 0.001. **E.** Invasive potential of SW480 and SW1116 transfected with the Scr siRNA or FOXK1 siRNA; **, P < 0.01. The experiments were repeated at least three times. Scale bars, 100 μm in A and B.

To assess the influence of FOXK1 on cancer cell migration and invasion, we first examined FOXK1 expression in primary CRC tissues by IHC. We found that were strongly present FOXK1-positive signals were present in the nucleus and cytoplasm of cancer cells (Figure 4A, primary cancer tissue) and tumor-associated stroma cells (TAS; Figure 3A, arrow).

Next, we detected the expression of FOXK1 in regional lymph nodes related with metastasis. In total, 29/32 of the metastatic tissues taken from lymph nodes highly expressed FOXK1 by means of IHC, as exemplified in two patients (Figure 4B). There was a correlation between the expression of FOXK1 in the primary lesion and metastasis to a regional lymph node.

Thirdly, we examined the role of FOXK1 in cell migration *in vitro*. We knock-down of FOXK1 cells using siRNA and confirmed this effect by Western blot analysis (Figure 4C). Cell migration was determined using a wound-healing assay. As shown in Figure 4D, RNAi- mediated repression of FOXK1 significantly suppressed the migration of SW480 and SW1116 cells. The migration index of FOXK1- knockdown cells was decreased by 58.3% and 62.5% at 36 and 60 h in SW480, respectively. Similar results were observed in SW1116 cells (Figure 4D). To examine the cell invasion activity *in vitro*, we used transwell inserts coated with Matrigel. After FOXK1 knockdown, the invasiveness of SW480 cells was decreased by 71.9% compared with the control cells (Figure 4E). Similar results were observed in SW1116 cells (Figure 4E).

These data indicated that FOXK1 knockdown suppressed CRC cell migration and invasion.

### Ectopic expression of FOXK1 induces EMT *in vitro*

EMT, a process by which tumor-associated epithelial cells obtain mesenchymal features, plays a critical role in tumor metastasis [27]. Several transcription factors that promote this process, including AP-1, NF-κB, snail, ZEB1, Sp1 and FOXA1, have been identified [28–32], however, the contributions of FOXK1 to EMT remain unclear.

**Figure 5:**
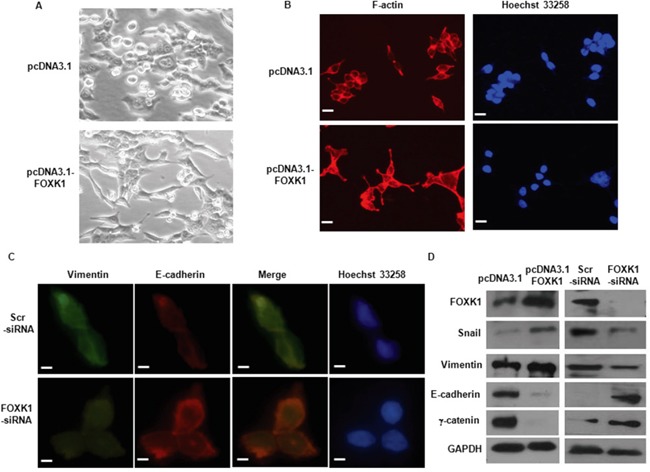
FOXK1 regulated epithelial-to-mesenchymal transition (EMT) *in vitro* **A.** Morphology of stable transfectants of SW480/Vector, SW480/FOXK1 as visualized under phase-contrast microscope. **B.** Stable transfectants stained with rhodamine-phallotoxin, with F-actin filaments visualized under fluorescent microscopy. **C.** E-cadherin expression correlates with increased in immunofluorescence analysis after SW480 FOXK1-siRNA. **D.** The EMT biomarkers, including E-cadherin, N-cadherin, vimentin, and fibronectin, were detected by Western blot. These experiments were repeated three times with identical findings. Scale bars represent 50 μm in A and B; 20μm in C.

To evaluate the role of FOXK1 in EMT, we first examined the morphologic features of SW480 cell. The stable transfectants of vector-transfected cells displayed a round or flat morphology with short cytoplasmic processes. However, pcDNA3.1-FOXK1- transfectants exhibited a spindle-like, fibroblastic morphology, which one of the main characteristics of EMT. Long or dendritic-like cytoplasmic processes were visible under a phase-contrast microscope (Figure 5A).

We then stained F-actin using phalloidin staining. Compared with the vector-expressing cells, FOXK1-overexpressing cells showed F-actin staining throughout the cytoplasm and at the rim zone of the protrusion. Moreover, filopodia and lamellipodia were identified as dynamic cellular features on the cell membrane surfaces that require actin polymerization and are involved in the invasion and metastasis of cancer cells (Figure 5B).

Third, we assessed the expression of EMT markers. E-cadherin was increased in immunofluorescence analysis after the knockdown of FOXK1 (Figure 5C). The down-regulation of mesenchymal markers (vimentin and snail) and the up-regulation of epithelial markers (E-cadherin and γ–catenin) were observed by Western blot after FOXK1-siRNA transfection (Figure 5D).

Together, these data indicate that FOXK1 can induce EMT in CRC cells.

### Knockdown of FOXK1 inhibited TGF-β1-induced EMT

**Figure 6:**
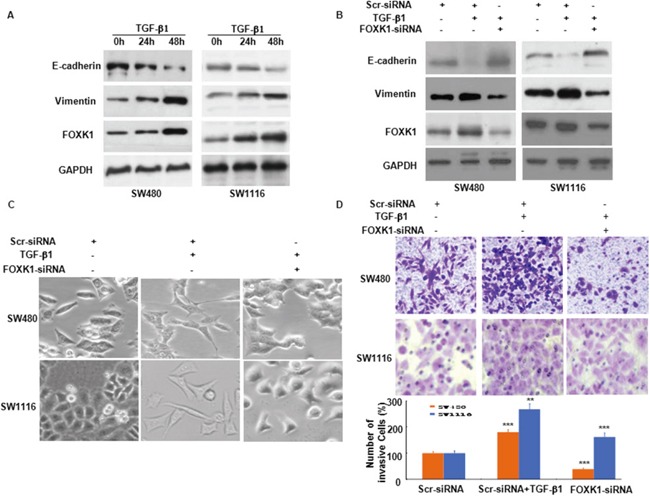
FOXK1 participates in TGF-β1-induced EMT **A.** Western blot of FOXK1, vimentin and E-cadherin in the indicated cells in response to treatment with 10 ng/mL TGF-β1 for 0, 24, and 48 hours. **B.** Twenty-four hours post-transfection of Scr siRNA or FOXK1 siRNA, the cells were treated with TGF-β1 (2 ng/ml) for an additional 48 h. FOXK1 expression was detected by Western blot. The expression of E-cadherin and vimentin was detected by Western blot, with GAPDH as the internal control. **C.** The morphology of SW480 and SW1116 cells was observed under an inverted microscope. **D.** Representative images and data of a transwell assay for SW480 and SW1116 cells. Each bar represents the mean ± SD. ***, P < 0.001 compared with that in the absence of TGF-β1. Compared with those transfected with FOXK1-siRNA and treated with TGF-β1: ***, P < 0.001 in SW480 and SW1116. All images are representative of three independent experiments with similar findings. Scale bars, 20 μm in C.

To determine the role of FOXK1 in EMT, we evaluated its response to the most potent EMT inducer, TGF-β1. As shown in Figure 6A, TGF-β1 induced FOXK1 expression in a time-dependent pattern. This induction also increased the expression of the mesenchymal marker, vimentin, whereas it decreased the expression of E-cadherin, an epithelial marker.

To explore the role of FOXK1 in TGF-β1-induced EMT and cell invasiveness, we transfected Scr-siRNA and FOXK1-siRNA into SW480 and SW1116 cells. A higher expression of epithelial markers and a reduced expression of mesenchymal markers were evident after FOXK1-siRNA expression treated with TGF-β1 for 48 h (Figure 6B). In addition, the results indicated that FOXK1-siRNA neutralized the influence of TGF-β1 on cell phenotype (Figure 6C). Coupled with the morphologic changes of EMT, the knockdown of FOXK1 decreased the invasive ability of tumor cells (Figure 6D).

The above results suggested that FOXK1 played an important role in TGF-β1-induced EMT in CRC.

### FOXK1 induced EMT and metastasis in CRC *in vivo*

**Figure 7:**
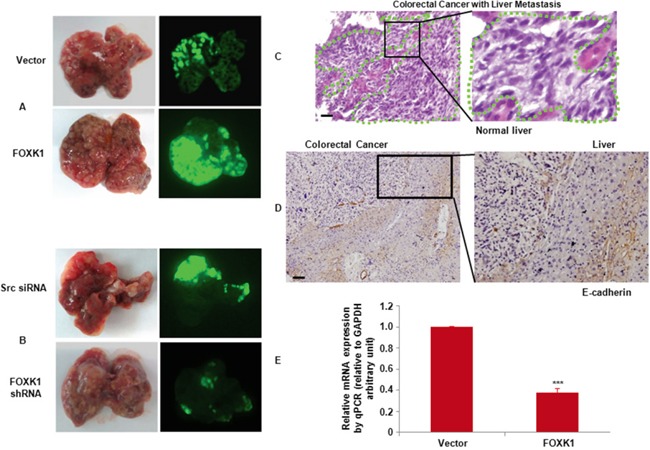
FOXK1 promoted tumour metastasis *in vivo* **A.** External whole-body fluorescence images of liver by injection of SW480/pEGFP-FOXK1 and SW480/pEGFP-N1 (Vector), or **B.** SW480/pEGFP-FOXK1 shRNA and SW480/pEGFP-src shRNA were obtained 30 days after spleen injection. The mice were sacrificed. **C.** Metastatic cancer tissues (arbitrary polygonal) were stained with H&E. **D.** E-cadherin expressions of the liver metastasis of colon carcinoma detected by IHC. **E.** Expression of E-cadherin-positive tumors derived from SW480 cells was determined by qRT-PCR; **, P < 0.01. Scale bars, 100 μm in C and D.

To further test the association of FOXK1 with metastasis, the role of FOXK1 in metastases was tested by injection of SW480/pEGFP-FOXK1 and SW480/pEGFP-N1 (Vector), or SW480/pEGFP-FOXK1 shRNA and SW480/pEGFP-src shRNA expressing with Green Fluorescent Protein (GFP) into nude mice. Thirty days after injection, the mice with FOXK1 overexpressing SW480 cells, but not vector SW480 cells, formed a variety of large metastatic nodules in livers (Figure 7A), whereas the mice with FOXK1 knockdown were in liver small nodules compared with those in the src shRNA (Figure 7B). The presence of liver metastases from CRC was confirmed by histological analysis (Figure 7C).

To further demonstrate whether FOXK1 is required for EMT, the explanted liver tissue showed that, compared with SW480/Vector the orthotopic implantation of SW480/FOXK1 expressing cells resulted in decreased E-cadherin expression in an IHC assay and qRT-PCR (Figure 7D & 7E).

Taken together, these results clearly indicated a critical role of FOXK1 in metastasis and the induction of EMT in CRC.

## DISCUSSION

In this study, we report on FOXK1, a gene that is over-expressed in various cancers, including CRC, acts as an important oncogene. We observed that FOXK1 played an important role in CRC progression and metastasis and was a novel unfavorable predictor biomarker for CRC patients. We also identified for the first time the role and functional pathway of FOXK1 in promoting EMT in colon cancer.

Forkhead/winged helix transcription factor family members have diverse functional roles during embryogenesis [1–3]. There is evidence that FOX protein has a central function in established cancers [11–13]. For example, FOXA1, the most extensively studied member of the family, was found to be up-regulated in many cancers, such as breast [32], bladder [33], prostate [34], glioma [35] and pancreatic [36] cancers. This protein contributes to many of the typical indications of cancer, including increased proliferation, resistance to cell death, and increased invasion and metastasis. Our study used tissue arrays to investigate FOXK1 expression profiles in various normal and cancer tissues. Normal skin, testis and kidney tissues were weakly positive for expression; 13 tissues were negative. However, all of the cancerous tissues were positive. Thus, FOXK1 may act as an oncogene.

Wang and colleagues reported that FOXK1 protein level are elevated in human CRC and positively regulate Wnt/β-catenin by translocating DVL into the nucleus, indicating its role as an oncogene [18]. However, FOXK1 expression and the relation to clinicopathological characteristics and prognosis of patients in human CRC remain largely unknown. In our study, we revealed a significant correlation between elevated FOXK1 expression and TNM stage, AJCC Stage, differentiation and lymph nodal metastasis. These strong correlations suggest that FOXK1 overexpression may promote tumor invasion and metastasis. Therefore, FOXK1 could be used as a biomarker to identify subsets of CRC with a more aggressive phenotype.

Kaplan-Meier analysis of the survival curves showed a significantly worse overall survival for patients whose tumors had high FOXK1 levels (log-rank test P=0.000), indicating that high FOXK1 tumor protein level is a marker of poor prognosis for patients with CRC. Chu showed FOXM1 over-expression plays a critical role in migration and invasion of CRC and the status of FOXM1 expression might be a prognostic factor for CRC patients [37]. Consistently, we have shown that FOXK1 expression was correlated with worse outcome and might be an independent prognostic factor for patients with CRC.

Epithelial-mesenchymal transition (EMT) is an orchestrated series of events in which cell-cell and cell-extracellular matrix (ECM) interactions are altered and the transition from epithelial phenotype to mesenchymal phenotype occurred [38, 39]. During cancer progression, advanced tumor cells frequently exhibit a conspicuous downregulation of epithelial markers and a loss of intercellular junctions, resulting in a loss of epithelial polarity and reduced intercellular adhesion. These alterations are often accompanied by increased cell motility and expression of mesenchymal-specific proteins [39, 40]. Therefore, EMT can promote hallmark features of carcinoma that correlates with poor histologic differentiation, destruction of tissue integrity, invasion and metastasis [40, 41]. Recently, the Forkhead transcription factor family, FOXQ1 and FOXM1 induce EMT and aggressiveness in human cancer [23, 24]. Consistently, we have shown that FOXK1 overexpression caused loss of epithelial polarity and the expression of EMT markers: loss of epithelial cell markers, such as E-cadherin and γ-catenin, and the upregulation of mesenchymal cell markers, including vimentin and snail. Stable transfectant of FOXK1 promoted migration, metastasis, and dissemination, thus facilitating tumor development and progression in CRC cells.

More importantly, our data have suggested that FOXK1 is also involved in and required for TGF-β–induced EMT [22, 29]. TGF-β signaling can act in either suppressing tumors or promoting tumors, depending on the course of cancer progression. In early tumor stages, TGF-β signaling suppresses tumor growth through inducing cell cycle arrest and apoptosis. But during the later stage of tumorigenesis, it promotes cancer invasion and metastasis through epithelial to mesenchymal transdifferentiation [42]. Mani et al. have shown that some members of the Forkhead-factor family play a role in mediating TGF-β-induced EMT. FOXC2 was overexpressed in invasive breast cancer cell lines [43]. Moreover, FOXQ1 promoted invasion and metastasis in colorectal cancer cells that had undergone EMT induced by TGF-β [34]. We found that induction of EMT in SW480 cells by TGF-β1 was associated with a significant increase in FOXK1 expression. TGF-β1 induced changes in morphology, significantly decreases E-cadherin expression and concomitantly increased vimentin expression, and promoted CRC cell invasiveness. Our findings suggest FOXK1 acts as a co-stimulator in TGF- β1-induced EMT in GC.

In conclusion, this study shows that FOXK1 is expressed at higher levels in 16 different types of solid tumor tissues. Moreover, we observed that FOXK1 is up-regulated in CRC, indicating its relationship with poor clinical outcomes. Furthermore, this study of FOXK1 is the first to indicate the contribution of EMT to tumor metastasis and the invasion of CRC cells *in vitro* and *in vivo*. Thus, our data imply that FOXK1 plays an important part in mediating CRC progression and may serve as a therapeutic target for CRC.

## MATERIALS AND METHODS

### Reagents, cell cultures and cell lines

Recombinant human TGF-β1 (240-B) was purchased from R&D Systems (Minneapolis, MN). Mouse anti-FOXK1, Snail, Vimentin, E-Cadherin, β-catenin and GAPDH were purchased from Santa Cruz Biotechnology (Santa Cruz, CA). FHC, HT-29, SW480, Lovo, SW1116, SW620, Colo205 and DLD1, cells were grown in RPMI 1640 containing 10% fetal bovine serum (Life Technologies, Monza, Italy), 1% glutamine (Life Technologies) and 1% penicillin/treptomycin (Life Technologies) in a humidified incubator at 37°C with an atmosphere of 5% CO_2_.

### Tissue multi-array (TMA)

TMAs were purchased from Alenabio Co, Ltd, Xian, Shanxi, China. (BN1002a and FDA800). TMAs with 16 types (3 spots for each) of normal and solid tumor tissues contains brain, breast, esophagus, kidney, liver, lung, cervix uteri, ovary, pancreas, prostate, skin, small intestine, stomach, testis, large intestine and rectum.

### Immunohistochemistry

Ninety-three surgical removal of CRC from 2005 to 2008 were selected from the Department of Surgery of Nanfang Hospital, Southern Medical University. The Ethics Committee of the Southern Medical University, China, approved our experimental protocols. Paraffin-embedded tissue blocks were cut into 5 μm sections and transferred to glass slides. The slides were deparaffinized with xylene, rehydrated with ethanol, washed and subjected to microwave retrieval in a citrate buffer. Sections were then immersed in 3% hydrogen peroxide to block endogenous peroxidase activity and incubated with the first antibodies followed by incubation with peroxidase-conjugated anti-rabbit secondary antibody (Dako) (1:100). The expression of FOXK1 was then visualized using 1 mg/ml 3, 3#-diaminobenzidine and counterstained with hematoxylin. Normal mouse IgG (Sigma) was used as an isotype control for anti-FOXK1 antibody to verify specificity of the staining. Histopathological analyses confirmed the malignant tissues. Tumor staging was defined according to the criteria for histological classification proposed by the International Union against Cancer (UICC). A tissue in which more than 10% of cancer cells stained positive was considered positive. For quantitative analysis, the ratio of positively stained cells to all tumor cells in five random areas at 200-fold magnification was recorded. Scoring of tissue slides was performed independently by two investigators; the percentage of positive cells was scored from 0 to 3 as follows: 0 (negative), <10% of cells stained; 1 (weak expression), 10–50% of cells stained; 2 (moderate expression), 50–75% of cells stained; 3 (strong expression), >75% of cells stained. An intensity score of ≥2 of FOXK1-positive cells was considered high expression, and an intensity score of 0-1 of FOXK1-positive cells was regarded as low expression.

### RNA isolation and quantitative real-time RT-PCR

Cells were harvested, and total RNA was extracted using Trizol Reagent (Gibco BRL, Gaithersburg, MD). RNA was reverse transcribed to cDNA by Thermoscript RT system reagent (Gibco BRL) in accordance with the manufacturer's instructions.

Quantitative real-time PCR was performed using an Applied Biosystems Sequence Detection System 7900 (ABI Prism 7900HT, Applied Biosystems Company, USA) with a 10- μl mixture composed of Power SYBR GREEN PCR Master Mix (Applied Biosystems, Foster City, CA), 500 nmol of each primer, and 300 ng of cDNA templates. The reactions were performed with initial denaturation at 95°C for 5 minutes followed by 60 cycles of 20 seconds at 94°C, 20 seconds at 60°C, and 40 seconds at 72°C. A final extension at 72°C for 5 minutes was included before a temperature ramp from 72°C to 95°C at 0.1°C/s with continuous fluorescent acquisition. Each cDNA sample was duplicated for each time of q-RT-PCR, and the average relative fold mRNA expression levels were determined using the 2^−ΔΔC^_t_ method, with GAPDH as the internal control. The primers used are listed in Supplementary Table S1 [44, 45].

### Transient siRNA transfection

Ablation of FOXK1 was performed by transfection with small interfering RNA (siRNA) duplex oligos, which was synthesized by Genepharma Company (Shanghai, China). Control siRNA (Scrambled RNA, Shanghai, China) and FOXK1-specific siRNA 1 (sense: 586- CCAUCAAGAUCCAGUUCAC (dTdT) -605, antisense: 605- GUGAACUGGAUCUUGAUGGdTdT -586 and FOXK1-specific siRNA 2 (sense: 889- GAGACAGCCCCAAGGAUGA (dTdT) -908, antisense: 908- UCAUCCUUGGGGCUGUCUC (dTdT) -889 were transfected using Lipofectamine 2000 (Invitrogen). Forty-eight hours after transfection and Western blot analysis were performed.

### Constructs and establishment of stable transfectants

To complementary DNA (cDNA) corresponding to the full-length FOXK1 was obtained by RT-PCR amplification of normal human testis cDNA with primers specific to FOXK1. The PCR aliquots were subcloned into mammalian expression vector pcDNA3.1 (Invitrogen, Carlsbad, CA).

To establish stable cell lines, cells transfected with empty pcDNA 3.1 vector and pcDNA3.1-FOXK1 were passaged at 1:15 (vol/vol) and cultured in RPMI 1640 medium supplemented with Geneticin (G418, Calbiochem, Darmstadt, Germany Canada) at 800 μg/ml for 4 weeks.

### Promoter activity assay for FOXK1

To elucidate the mechanism of how FOXK1 regulates the expression of Survivin, Cyclin D1, AP-1, ZEB1 and TERT, promoter assays were performed. For the promoter assay, we used the human Survivin, Cyclin D1, AP-1, ZEB1 and TERT and promoter-driven luciferase (Luc) reporter plasmid pGL3, which contained a 3.0-kbp fragment upstream of these 5 genes. Plasmid integrity was confirmed by DNA sequencing. The cells were cotransfected using the Lipofectamine 2000 solution (Invitrogen) with reporter plasmids and the pcDNA3.1- FOXK1 vector. After 36 h of incubation, the cells were harvested, and Luc activity in the cellular lysates was measured with a luminometer. Enzyme activities were normalized. To the control, which was transfected only reporter plasmid. The firefly luciferase activity value was normalized to the Renilla activity value. Promoter activity was presented as the fold induction of the relative luciferase unit (RLU) compared with the basic vector control. RLU = value of the firefly luciferase unit/value of the Renilla luciferase unit. All treatments were performed in triplicate for each single experiment.

### Western blot analysis and immunofluorescence

For western blot analysis, 48 hours after transfection, cells were harvested and lysed in lysis buffer [10 mmol/L Tris-HCl (pH 7.4), 1% SDS, 10% glycerol, 5 mmol/L MgCl2, 1 mmol/L phenylmethylsulfonyl fluoride, 1 mmol/L sodium orthovanadate, 5 μg/mL leupeptin, and 21 μg/mL aprotinin]. A total of 30 μg of protein lysates were separated by SDS-PAGE and transferred onto a PVDF membrane. The dilution of primary antibodies was according to the company's recommendation. Proteins were visualized using the enhanced chemiluminescence detection system.

Cells grown in cover glass were fixed with 4% paraformaldehyde and the nonspecific bindings were block by incubation with 1% bovine serum albumin. The glasses were probed with the first antibodies followed by TR- or fluorescein isothiocyanate-conjugated second antibodies. After mounting, the slips were visualized under an Olympus CKX 41 fluorescence microscope (Olympus Optical Co., Ltd., Tokyo, Japan).

For staining of F-actin, cells were washed with PBS and fixed in methanol/acetone (1:1) for 5 min on ice, and incubated with rhodamine-conjugated phallotoxin (5 U/mL, Molecular Probes) in PBS at a 1:40 dilution for 1 h. Coverslips were washed, mounted, and visualized using fluorescence microscope. Nuclei were stained with 1 μg/mL Hoechst 33258 and cells were analyzed using fluorescence microscope.

### Cell migration and invasion assays

The small interfering RNA (siRNA) transfection were seeded at 1 × 10^6^ per well into six-well plates and grown to confluence for 24 h. The monolayer was wounded with a pipette tip and cells detached upon wounding were carefully rinsed off. Media was changed to remove cell debris and the cells were cultured in presence of 10 μg/ml mitomycin C to inhibit cell proliferation. Photographs were taken 60 h later. Cell invasion was assessed using Matrigel invasion chamber (BD Biosciences) as per the protocol provided by the manufacture. Briefly, cells 24 h post the siRNA transfection were resuspended in serum free media. 5 × 10^5^ of cells were placed to each Transwell membrane filter inserts with the lower chamber filled with 600 μl of complete medium and incubated for additional 24 h. Invasive cells were stained with 0.2% of crystal violet and counted under a microscope. The average number of cells in five fields per membrane was counted in triplicate inserts. The invasion index was expressed as the percentage of test cells to that of control cells or treatments.

### Construction of lentivirus vectors with FOXK1 short hairpin RNA

To investigate further the effect of small interfering RNA (siRNA)-induced knock-down of FOXK1 expression on the *in vivo* tumor metastasis of colorectal cancer, a FOXK1-RNAi lentiviral vector (pGCSIL-FOXK1-shRNA) was constructed (Shanghai GeneChem Co, Ltd, Shanghai, China). Double-stranded oligonucleotides encoding human FOXK1-vshRNA (NM_00103716; CCGGGAGACAGCCCCAAGGATGATCAAGAGTCATCCTTGGGGCT GTCTCTTTTTG) were annealed and inserted into the short hairpin RNA (shRNA) expression vector pGCSIL-GFP (Shanghai GeneChem Co, Ltd). A GFP-lentiviral vector (pGCSIL-GFP) was used as a negative control. Clone identity was verified by sequencing.

Recombinant lentiviral vector was produced by co-transfecting HEK293T cells with lentiviral expression vector and packing plasmid mix using Lipofectamine™ 2000, according to the manufacturer's instructions. Infectious lentiviral particles were harvested at 48 h post-transfection, then filtered through 0.45 μm cellulose acetate filters. The virus was concentrated, then, titer was determined by serial dilution on 293 T cells. For lentivirus transduction, SW480 (SW1116) cells were subcultured at 1 ×10^5^ cells/well into 6-well culture plates. Cells were transducted with FOXK1-shRNA-expressing (FOXK1 shRNA) or src-shRNA-expressing lentivirus at a multiplicity of infection (MOI) of 50 (moi 100). Cells were harvested at 72 h after infection and the transduction efficiency was evaluated by counting the percentage of GFP-positive cells.

### *In vivo* metastasis assays

Four- to 6-week-old BALB/C-nu/nu nude mice were obtained from the Laboratory Animal Unit, Southern Medical University, China. To evaluate the metastatic potential to liver of cancer cells *in vivo*, 5×10^6^ SW480/pEGFP-N1, SW480/pEGFP- FOXK1, SW480/pEGFP-src shRNA and SW480/pEGFP-FOXK1 shRNA cells were inoculated into the dorsal subcostal incision to expose the spleen (n = 3 for each group). The volume (50 μL) of tumor cell suspension was injected slowly into the spleen using a 25-gauge needle. One month later, the mice were sacrificed; the individual organs were removed and assessed using the *In-Vivo* F Imaging System (Kodak). The metastatic tissues were analyzed with H&E, IHC staining and qRT-PCR assay.

### Statistical analyses

Statistical analyses were performed using the SPSS 17.0. Correlation between FOXK1 expression and clinicopathological characteristics were evaluated by Chi-square test. The survival rates after tumor removal were calculated by the Kaplan-Meier method, and the differences in survival curves were analyzed by log-rank tests. The χ^2^, Fisher exact probability, and Student's t tests were used for comparison between groups. P < 0.05 was considered significant.

## SUPPLEMENTARY FIGURES AND TABLE



## Abbreviations

EMT: epithelial-to-mesenchymal transition
ATCC: American Type Culture Collection
qRT-PCR: quantitative real-time -PCR
CRC: colorectal cancer
IHC: immunohistochemistry
TMA: tissue multi-array
Fox: the forkhead box
FOXK1: the forkhead box k1
AJCC: American Joint Committee on Cancer
siRNA: small interfering RNA
RLU: relative luciferase unit
TAS: tumor-associated stroma cells
MOI: multiplicity of infection

## References

[1] Katoh M, Katoh M (2004). Human FOX gene family. Int J Oncol.

[2] Murakami H, Aiba H, Nakanishi M, Murakami-Tonami Y (2010). Regulation of yeast forkhead transcription factors and FoxM1 by cyclin-dependent and polo-like kinases. Cell Cycle.

[3] Yang Y, Hou H, Haller EM, Nicosia SV, Bai W (2005). Suppression of FOXO1 activity by FHL2 through SIRT1-mediated deacetylation. EMBO J.

[4] Foucher I, Volovitch M, Frain M, Kim JJ, Souberbielle JC, Gan L, Unterman TG, Prochiantz A, Trembleau A (2002). Hoxa5 overexpression correlates with IGFBP1 upregulation and postnatal dwarfism: evidence for an interaction between Hoxa5 and Forkhead box transcription factors. Development.

[5] Gaudet J, Mango SE (2002). Regulation of organogenesis by the Caenorhabditis elegans FoxA protein PHA-4. Science.

[6] Wang X, Quail E, Hung NJ, Tan Y, Ye H, Costa RH (2001). Increased levels of forkhead box M1B transcription factor in transgenic mouse hepatocytes prevent age-related proliferation defects in regenerating liver. Proc Natl Acad Sci U S A.

[7] Foucher I, Volovitch M, Frain M, Kim JJ, Souberbielle JC, Gan L, Unterman TG, Prochiantz A, Trembleau A (2002). Hoxa5 overexpression correlates with IGFBP1 upregulation and postnatal dwarfism: evidence for an interaction between Hoxa5 and Forkhead box transcription factors. Development.

[8] Clevidence DE, Overdier DG, Tao W, Qian X, Pani L, Lai E, Costa RH (1993). Identification of nine tissue-specific transcription factors of the hepatocyte nuclear factor3/forkhead DNA-binding -domain family. Proc Natl Acad Sci U S A.

[9] Gao N, Ishii K, Mirosevich J, Kuwajima S, Oppenheimer SR, Roberts RL, Jiang M, Yu X, Shappell SB, Caprioli RM, Stoffel M, Hayward SW, Matusik RJ (2005). Forkhead box A1 regulates prostate ductal morphogenesis and promotes epithelial cell maturation. Development.

[10] Williamson EA, Wolf I, O'Kelly J, Bose S, Tanosaki S, Koeffler HP (2006). BRCA1 and FOXA1 proteins coregulate the expression of the cell cycle-dependent kinase inhibitor p27(Kip1). Oncogene.

[11] Coffer PJ, Burgering BM (2004). Forkhead-box transcription factors and their role in the immune system. Nat Rev Immunol.

[12] Müller SM, Terszowski G, Blum C, Haller C, Anquez V, Kuschert S, Carmeliet P, Augustin HG, Rodewald HR (2005). Gene targeting of VEGF-A in thymus epithelium disrupts thymus blood vessel architecture. Proc Natl Acad Sci U S A.

[13] Teh MT, Wong ST, Neill GW, Ghali LR, Philpott MP, Quinn AG (2002). FOXM1 is a downstream target of Gli1 in basal cell carcinomas. Cancer Res.

[14] Garry DJ, Yang Q, Bassel-Duby R, Williams RS (1997). Persistent expression of MNF identifies myogenic stem cells in postnatal muscles. Dev Biol.

[15] Garry DJ, Meeson A, Elterman J, Zhao Y, Yang P, Bassel-Duby R, Williams RS (2000). Myogenic stem cell function is impaired in mice lacking the forkhead/winged helix protein MNF. Proc Natl Acad Sci U S A.

[16] Yang Q, Kong Y, Rothermel B, Garry DJ, Bassel-Duby R, Williams RS (2000). The winged-helix/forkhead protein myocyte nuclear factor beta (MNF-beta) forms a co-repressor complex with mammalian sin3B. Biochem J.

[17] Huang JT, Lee V (2004). Identification and characterization of a novel human FOXK1 gene in silico. Int J Oncol.

[18] Wang W, Li X, Lee M, Jun S, Aziz KE, Feng L, Tran MK, Li N, McCrea PD, Park JI, Chen J (2015). FOXKs promote Wnt/β-catenin signaling by translocating DVL into the nucleus. Dev Cell.

[19] Gumireddy K, Li A, Gimotty PA, Klein-Szanto AJ, Showe LC, Katsaros D, Coukos G, Zhang L, Huang Q (2009). KLF17 is a negative regulator of epithelial- mesenchymal transition and metastasis in breast cancer. Nat Cell Biol.

[20] Yang MH, Wu MZ, Chiou SH, Chen PM, Chang SY, Liu CJ, Teng SC, Wu KJ (2008). Direct regulation of TWIST by HIF-1alpha promotes metastasis. Nat Cell Biol.

[21] Okada T, Sinha S, Esposito I, Schiavon G, López-Lago MA, Su W, Pratilas CA, Abele C, Hernandez JM, Ohara M, Okada M, Viale A, Heguy A, Socci ND, Sapino A, Seshan VE, Long S, Inghirami G, Rosen N, Giancotti FG (2015). The Rho GTPase Rnd1 suppresses mammary tumorigenesis and EMT by restraining Ras-MAPK signalling. Nat Cell Biol.

[22] Fan Y, Shen B, Tan M, Mu X, Qin Y, Zhang F, Liu Y (2014). TGF-β-induced upregulation of malat1 promotes bladder cancer metastasis by associating with suz12. Clin Cancer Res.

[23] Huang C, Xie D, Cui J, Li Q, Gao Y, Xie K (2014). FOXM1c promotes pancreatic cancer epithelial-to-mesenchymal transition and metastasis via upregulation of expression of the urokinase plasminogen activator system. Clin Cancer Res.

[24] Xia L, Huang W, Tian D, Zhang L, Qi X, Chen Z, Shang X, Nie Y, Wu K (2014). Forkhead box Q1 promotes hepatocellular carcinoma metastasis by transactivating ZEB2 and VersicanV1 expression. Hepatology.

[25] Belguise K, Guo S, Sonenshein GE (2007). Activation of FOXO3a by the green tea polyphenol epigallocatechin-3-gallate induces estrogen receptor alpha expression reversing invasive phenotype of breast cancer cells. Cancer Res.

[26] Christofori G (2006). New signals from the invasive front. Nature.

[27] Zhang W, Jiang B, Guo Z, Sardet C, Zou B, Lam CS, Li J, He M, Lan HY, Pang R, Hung IF, Tan VP, Wang J, Wong BC (2010). Four-and-a-half LIM protein 2 promotes invasive potential and epithelial-mesenchymal transition in colon cancer. Carcinogenesis.

[28] Du J, Yang S, An D, Hu F, Yuan W, Zhai C, Zhu T (2009). BMP-6 inhibits microRNA-21 expression in breast cancer through repressing deltaEF1 and AP-1. Cell Res.

[29] Brandl M, Seidler B, Haller F, Adamski J, Schmid RM, Saur D, Schneider G (2010). IKK(α) controls canonical TGF(ß)-SMAD signaling to regulate genes expressing SNAIL and SLUG during EMT in panc1 cells. J Cell Sci.

[30] Zhang W, Wang J, Zou B, Sardet C, Li J, Lam CS, Ng L, Pang R, Hung IF, Tan VP, Jiang B, Wong BC (2011). Four and a half LIM protein 2 (FHL2) negatively regulates the transcription of E-cadherin through interaction with Snail1. Eur J Cancer.

[31] Zhang W, Shi XP, Peng Y, Wu MY, Zhang P, Xie RY, Wu Y, Yan QQ, Liu SD, Wang J (2015). HIF-1α promotes epithelial-mesenchymal transition and metastasis through direct regulation of ZEB1 in colorectal cancer. PLoS One.

[32] Jungert K, Buck A, von Wichert G, Adler G, König A, Buchholz M, Gress TM, Ellenrieder V (2007). Sp1 is required for transforming growth factor-beta-induced mesenchymal transition and migration in pancreatic cancer cells. Cancer Res.

[33] Badve S, Turbin D, Thorat MA, Morimiya A, Nielsen TO, Perou CM, Dunn S, Huntsman DG, Nakshatri H (2007). FOXA1 expression in breast cancer--correlation with luminal subtype A and survival. Clin Cancer Res.

[34] Fan DM, Feng XS, Qi PW, Chen YW (2014). Forkhead factor FOXQ1 promotes TGF-β1 expression and induces epithelial-mesenchymal transition. Mol Cell Biochem.

[35] Reddy OL, Cates JM, Gellert LL, Crist HS, Yang Z, Yamashita H, Taylor JA, Smith JA, Chang SS, Cookson MS, You C, Barocas DA, Grabowska MM, Ye F, Wu XR, Yi Y, Matusik RJ, Kaestner KH, Clark PE, DeGraff DJ (2015). Loss of FOXA1 Drives Sexually Dimorphic Changes in Urothelial Differentiation and Is an Independent Predictor of Poor Prognosis in Bladder Cancer. Am J Pathol.

[36] Mirosevich J, Gao N, Gupta A, Shappell SB, Jove R, Matusik RJ (2006). Expression and role of Foxa proteins in prostate cancer. Prostate.

[37] Chu XY, Zhu ZM, Chen LB, Wang JH, Su QS, Yang JR, Lin Y, Xue LJ, Liu XB, Mo XB (2012). FOXM1 expression correlates with tumor invasion and a poor prognosis of colorectal cancer. Acta Histochem.

[38] Przybylo J.A, Radisky DC (2007). Matrix metalloproteinase-induced epithelial mesenchymal transition: tumor progression at Snail's pace. Int. J. Biochem Cell Biol.

[39] Turley EA, Veiseh M, Radisky DC, Bissell MJ (2008). Mechanisms of disease: epithelial-mesenchymal transition-does cellular plasticity fuel neoplastic progression?. Nat. Clin. Pract. Oncol.

[40] Huber MA, Kraut N, Beug H (2005). Molecular requirements for epithelial- mesenchymal transition during tumor progression. Curr. Opin. Cell Biol.

[41] Bates RC, Mercurio AM (2005). The epithelial-mesenchymal transition (EMT) and colorectal cancer progression. Cancer Biol. Ther.

[42] Yan Q, Zhang W, Wu Y, Wu M, Zhang M, Shi X, Zhao J, Nan Q, Chen Y, Wang L, Cheng T, Li J, Bai Y, Liu S, Wang J (2015). KLF8 promotes tumorigenesis, invasion and metastasis of colorectal cancer cells by transcriptional activation of FHL2. Oncotarget.

[43] Mani SA, Yang J, Brooks M, Schwaninger G, Zhou A, Miura N, Kutok JL, Hartwell K, Richardson AL, Weinberg RA (2007). Mesenchyme Forkhead 1 (FOXC2) plays a key role in metastasis and is associated with aggressive basal-like breast cancers. Proc Natl Acad Sci U S A.

[44] Li JC, Yang XR, Sun HX, Xu Y, Zhou J, Qiu SJ, Ke AW, Cui YH, Wang ZJ, Wang WM, Liu KD, Fan J (2010). Up-regulation of Krüppel-like factor 8 promotes tumor invasion and indicates poor prognosis for hepatocellular carcinoma. Gastroenterology.

[45] Bao R, Connolly DC, Murphy M, Green J, Weinstein JK, Pisarcik DA, Hamilton TC (2002). Activation of cancer-specific gene expression by the survivin promoter. J Natl Cancer Inst.

