# Supplementary material for: Oncogene FOXK1 enhances invasion of colorectal carcinoma by inducing epithelial-mesenchymal transition
Source: Oncotarget. 2016 May 19;7(32):51150–62. doi: 10.18632/oncotarget.9457 (PMC5239465; doi:10.18632/oncotarget.9457)
Supplement: Supplementary file 1 [file oncotarget-07-51150-s001.pdf]

Oncogene FOXK1 enhances invasion of colorectal carcinoma by inducing epithelial-mesenchymal transition

SUPPLEMENTARY FIGURES AND TABLE

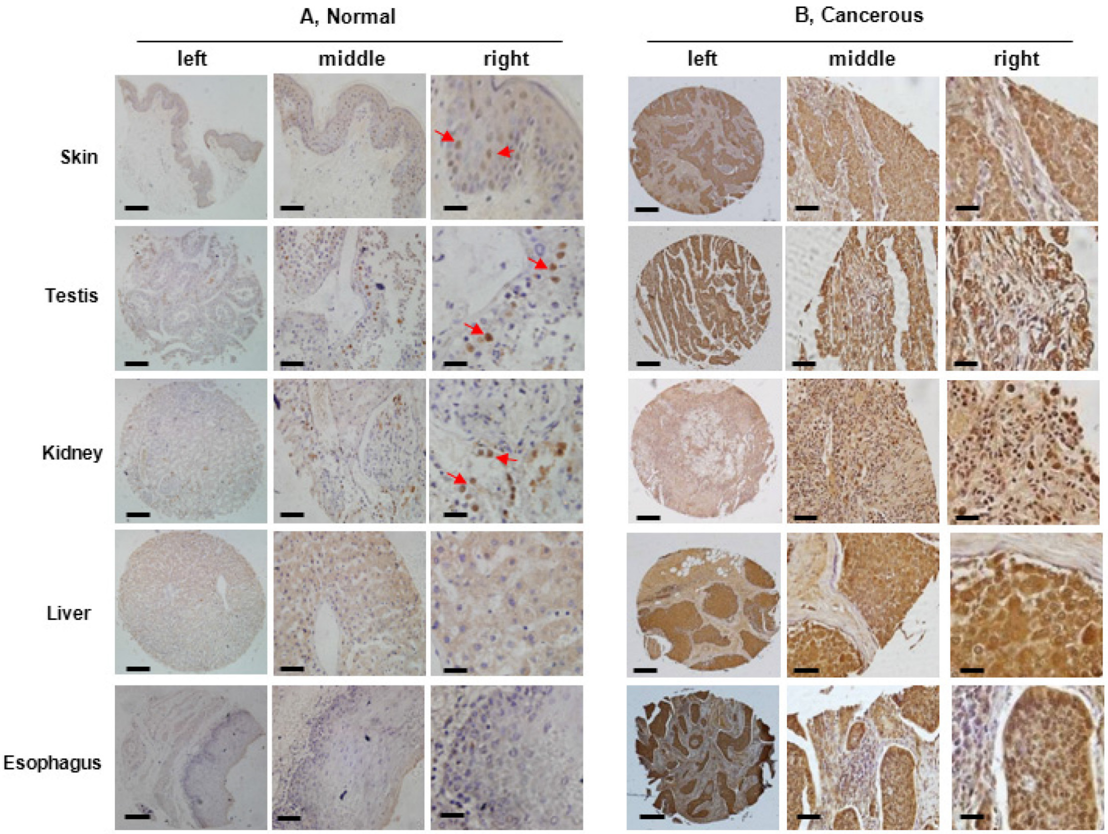

Supplementary Figure 1 (*continued*):

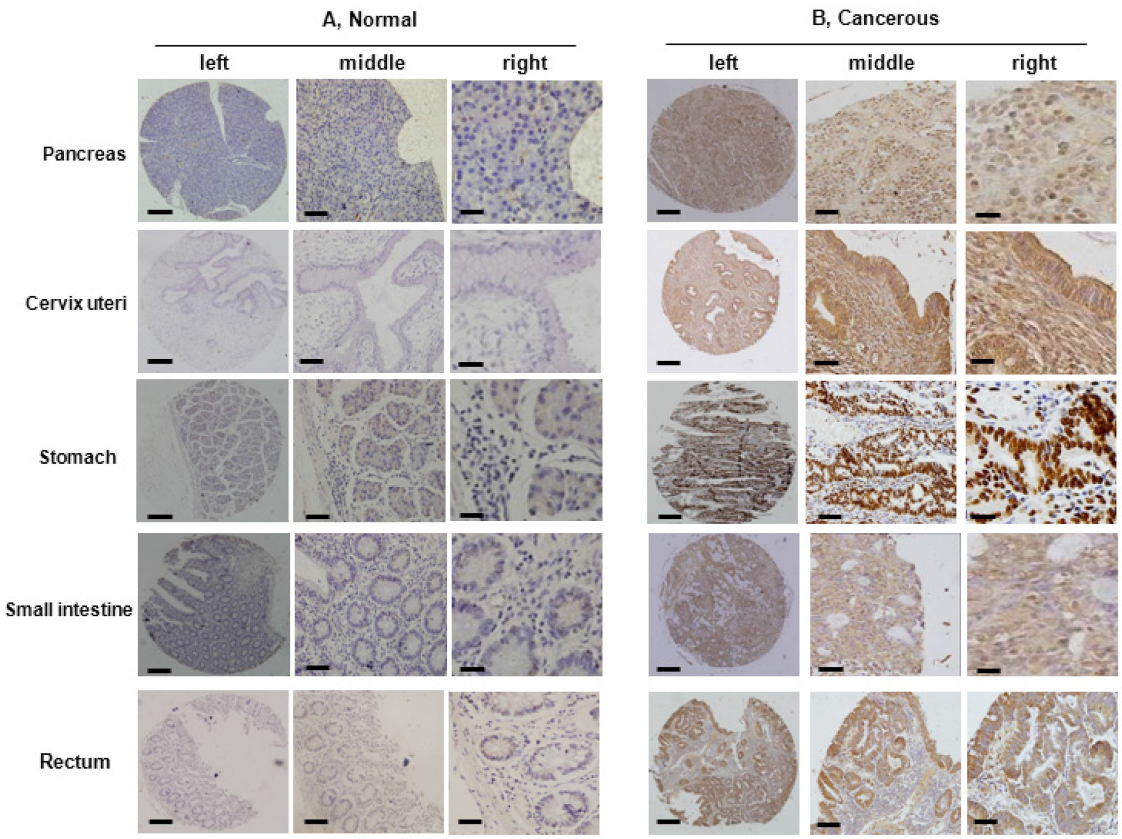

Supplementary Figure 1 (*continued*):

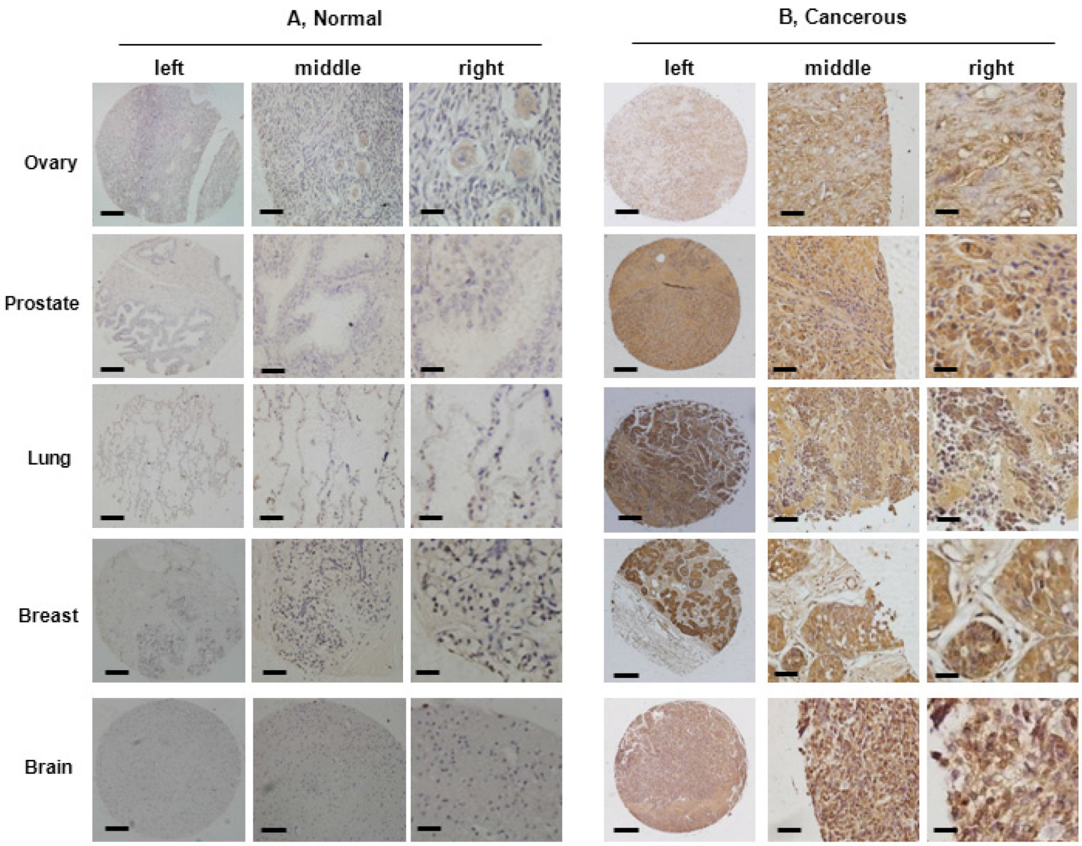

Supplementary Figure 1: FOXK1 expression in normal A. and malignant human tissues **B.** FOXK1 expression in normal and malignant human tissues was detected by immunohistochemistry. Scale bars represent 100 μm left panels; 20 μm middle panels and 5μm right panels of A, B and C.

Supplementary Table 1: The primers used are listed

FOXK1	F: 5′- ACACGTCTGGAGGAGACAGC -3′
	R: 5′- GAGAGGTTGTGCCGGATAGA -3′
Survivin	F: 5′- TGTCTTGAAAGTGGCACCAG -3′
	R: 5′- GCCTTCTTCCTCCCTCACTT -3′
Cyclin D1	F: 5′- CGTGGCCTCTAAGATGAAGG -3′
	R: 5′- CTGGCATTTTGGAGAGGAAG -3′
Ap-1	F: 5′- CCCCAAGATCCTGAAACAGA -3′
	R: 5′- CCGTTGCTGGACTGGATTAT -3′
Zeb1	F: 5′- CGCTTTACCTCTCTGAAAGAACA -3′
	R: 5′- TTACACCCAGACTGCGTCAC -3′
Tert	F: 5′- GCGTTTGGTGGATGATTTCT -3′
	R: 5′- TGTTCACCTGCAAATCCAGA -3′
GAPDH	F: 5′- GTCAACGGATTTGGTCGTATTG -3′
	R: 5′- CTCCTGGAAGATGGTGATGGG -3′
